# Assessment of breast cancer risk perception, knowledge, and breast self-examination practices among market women in Owo, Ondo State, Nigeria

**DOI:** 10.1186/s12905-023-02711-7

**Published:** 2023-10-27

**Authors:** Mujidat Awogbayila, Olayinka Onasoga, Umar Jibril, Funmilayo Oluwafemi, Edidiong Orok

**Affiliations:** 1 Department of Nursing Services, Afe Babalola University, Ado-Ekiti, Ekiti State Nigeria; 2 Department of Nursing Science, University of Ilorin, Ilorin, Kwara State Nigeria; 3 Department of Public Health, College of Medicine and Health Sciences, Afe Babalola University, Ado-Ekiti, Ekiti State Nigeria; 4 Department of Clinical Pharmacy and Public Health, Afe Babalola University, Ado-Ekiti, Ekiti State Nigeria

**Keywords:** Breast cancer, Risk perception, Knowledge, Breast self-examination, Practice, Market women, Ondo State, Nigeria

## Abstract

**Background:**

Breast cancer (BC) is the leading cause of cancer death among women worldwide, and its incidence is increasing, particularly in low-medium-income countries (LMICs). Evidence shows that breast self-examination (BSE) is culturally acceptable, religiously friendly and inexpensive. This study assessed BC risk perception, knowledge and breast self-examination practices among market women in Ondo State, Nigeria.

**Methods:**

A descriptive cross-sectional survey was conducted among market women in 3 selected markets in Owo. A semi-structured interview-based questionnaire was used to collect data. The instrument consisted of five sections based on the objective of the study. Selection of the study participants was done using a multistage sampling technique. The test–retest method was used to determine the reliability of the instrument. Participants knowledge and practices were categorised into good (≥ 50% total score) and poor (< 50% total score) while risk perception was grouped into high (≥ 50% total score) and low (< 50% total score).Data were analysed using descriptive and inferential statistics at a *p* value < 0.05 for determining statistical significance.

**Results:**

A total of 335 respondents completed the study and the mean age ± S.D. was 37.19 ± 9.19 years (range: 18–65 years). 47.5% of respondents were Christian while 60.0% of the respondents were married. 15.5% had tertiary education, and more than two-thirds were from the Yoruba tribe. More than two-thirds (78.5%) of the participants stated that they practiced breast self-examination, while 58.5% reported to have been taught how to perform BSE. 75.8% agreed that the use of injectable contraception or oral pills can result in BC, while 75.8% also agreed that women of reproductive age are at risk of BC. Less than 50% mentioned that they were uncomfortable taking off clothes in front of health professionals during the examination. The perceived risk of BC showed that 221 (65.97%), and 114 (34.03%) of respondents had high, and low levels of perceived risk of BC, respectively. However, 184 (54.93%) and 151 (45.07%) of the respondents had good and poor knowledge. The majority (139, 41.49%) of the respondents had poor BSE practice. Age (*p* = 0.023), educational qualifications (*p* < 0.001), average income per month (*p* < 0.001) and ethnicity (*p* =  < 0.001) were statistically associated with knowledge of breast self-examination while religion (*p* = 0.02), marital status (*p* = 0.01), educational qualification (*p* = 0.001) and distance from facility (*p* = 0.009) were statistically associated with perceived risk of BC. Participants’ educational qualification (*p* = 0.006) and ethnicity (*p* = 0.013) were statistically associated with practice of BSE. Good knowledge was also identified as a significant predictor of good practice of participants among the women (95%CI: 4.574 (2.841–7.365), *p* < 0.001).

**Conclusion:**

This study identified high level of perception, good knowledge and good practice of BSE among majority of the market women in Owo Town. Interventions and extensive health education on BSE with the aim of creating positive awareness and understanding of BSE among the population should be encouraged.

## Background of the study

Breast cancer (BC) is a cancerous growth arising in the lining cells of the ducts or lobules in the glandular tissue of the breast. It is a non-communicable and serious disease seen in women and the second most common cancer worldwide and a leading cause of cancer death in less developed countries of the world [1]. Breast self-examination (BSE) is a simple, cost-effective strategy for the early detection of any breast anomalies, especially in resource-poor countries such as Nigeria. It is a non-invasive and non-hazardous approach to increase breast health awareness and thus potentially allows for early detection of cancer of the breast, which is a major public health issue globally [2]. Appropriate, prompt and regular breast self-examination is a potent approach to reducing mortalities and morbidities associated with BC among women [3].

BC is the most common type of cancer affecting women in the developing and developed countries [4]. Globally, the BC burden is increasing; nearly 1.68 million new cases of BC were diagnosed in 2012, and almost 2.1 million new cases were diagnosed in 2018, while in 2020, 2.26 million cases of BC were diagnosed globally [3]. In Africa, breast cancer caused 74,072 deaths in 2018, and 168,690 cases were diagnosed [5]. However, the most worrisome part is that Nigeria ranks at the top in the absolute burden of BC cases, with 26,310 cases diagnosed and 11,564 deaths recorded in 2018, followed by Egypt [5].

In Nigeria, incomplete data and information from cancer registries make the burden of cancer unknown. Nevertheless, approximately 100,000 new cancer cases occur annually, with a high fatality ratio [6]. Historically, in Nigeria, the number of cases of cancer was low but is now on the increase due to changes in lifestyle and urbanization.

Risk perception could be viewed as a powerful force driving women to utilize screening and preventive measures; nevertheless, reports from previous studies indicated that women usually perceive breast cancer risk incorrectly [7]. Women’s risk perception about cancer will determine their use of diagnostic tools such as clinical breast examination (CBE), breast self-examination and mammography [8]. Breast self-examination is vital in detecting abnormalities in the breast; it remains a powerful tool in developing countries for detecting abnormalities in the breast, and breast self-examination provides opportunities for females to be conversant with their breast, thereby reporting any changes [7].

As devitalizing as cancer of the breast is, a large number of females in Nigeria have poor knowledge about it [9]. Moreover, Nigerian women’s attitudes and practices regarding breast self-examination remain poor, thereby leading to untimely death. The poor attitude of seeking health care portrayed by the women in Nigeria can be attributed to economic, religious and sociocultural factors [9].

In Nigeria, national screening programmes are not properly established; clinicians and women still rely on breast examination for early detection of BC. Previous studies reported a low level of BC risk perception leading to a poor response to breast screening measures [10, 11]. This high incidence of BC and the late presentation to the hospital with advanced stage of the disease when little or no benefit can be derived from therapy necessitates the need for early screening methods since it aids early initiation of treatment, thereby reducing mortality [12].

Female market traders engage in physically demanding roles and their marketing activities are linked to a sedentary lifestyle. Sedentary habits are prevalent in jobs that involve extended periods of inactivity and have been connected to obesity and related non-communicable diseases including breast cancer [13]. These women are susceptible to sedentary behavior, which puts them at risk of these diseases which is why this study was carried put among market women. An increased incidence of BC has been reported by the state government in Ondo State. An anecdotal report shows that poor perception of BC risk among these women has contributed to poor practice of breast self-examination and late presentation of BC to the hospital. Furthermore, the researcher’s experience in the clinical area has shown that despite the various health talks, there is still poor perception of BC risk and practice of breast self-examination. Based on the above, the authors decided to assess BC risk perception and breast self-examination practice among market women in Owo, Ondo state. Therefore, the main aim of this study was to evaluate BC risk perception, knowledge and practice of breast self-examination among market women in Owo, Ondo State.

### Research design

A descriptive cross-sectional study design using a quantitative approach was employed for this study.

### Research setting

This research was carried out in some markets in Owo, Ondo state. The markets chosen for this study are the three largest markets in Owo, which are the Oja Oba market, Ehinogbe market, and Iyere market out of five markets in Owo town. The Oba market consists of 63 locked-up shops and 240 open shops with an expanse open space. The Oja Oba is a daily market, and an average of two thousand nine hundred people sell in the market daily. The Ehinogbe market, otherwise known as the Anaye market, is located at Ehinogbe, Owo, founded over a century ago. It contains 200 stalls and open space for sales; presently, the lock-up shops are under construction. An average of three hundred people sell in the market daily. The Iyere market is another market located in Iyere quarters, Owo, which has an average of 150 traders on market days [14].

### Target population

The target population was market women positioned to sell commodities in allotted shops inside the market.

### Inclusion criteria and exclusion criteria

Women within the age range of eighteen and sixty-five years who gave consent to participate were included while those who did not give consent were excluded.

### Sample size determination

Sample size was calculated using Fisher’s formula [15].

$$n=\frac{{z}^{2}pq}{{d}^{2}}$$

n = sample size

z = the standard normal deviation, which corresponds to the 95% confidence level = 1.96.

p = estimate of key proportion = 27.3% derived from a similar study by Usman et al. (2020) [16].

$$q=1-p$$

d = degree of accuracy desired = 0.05

$$\begin{array}{c}n=\;\frac{{1.96}^2\;\times\;0.273\;\times\;0.727}{{0.05}^2}\\=\;304.98\;=305\end{array}$$

10% attrition rate = 30. Therefore, sample size = 305 + 30 = 335.

A total of 335 respondents were included in the study.

### Sampling technique

A multistage sampling method was used for this study. Two stages were employed during sampling.

#### Stage 1

Three markets were selected using simple random sampling from a total of six markets in Owo town. The markets were the Oba market, Ehinogbe market and Iyere market. Thereafter, the population size of market women was collected from the leader of the market women association of each market to calculate the number of women to be sampled in each market. The number of respondents in each market was determined by simple proportion (Table 1).

**Table 1:** Proportion of sample size

Selected Market	Estimated population	Number of respondents to be sampled	Approximate number to be sampled
Iyere market	150	150 ÷ 3350 × 335 = 14.99	15
Anaye (Ehinogbe)	300	300 ÷ 3350 × 335 = 29.99	30
Oja Oba market	2900	2900 ÷ 3350 × 335 = 290	290
Total	3350	335	335

#### Stage 2

From each market, the women were grouped into clusters of women. A total of 15 clusters were identified in each market and 10 clusters were selected based on the location of the shops in the market using simple random sampling. A total estimated population of 3350 from the three selected markets (Iyere market:150; Anaye (Ehinogbe): 300; Oja Oba market: 2900) was used as a sampling frame (Table 1).

### Instrument for data collection

A semi-structured questionnaire tagged “Breast Cancer Risk Perception and Breast Self- Examination Questionnaire” (BCRPBSE) written in English and translated into Yoruba was developed after rigorous revision of literature on the subject. The BCRPBSE contained five sections labelled A-E.

#### Section A

This measured the sociodemographic data of the respondents. It contained seven (7) items, such as age, religion, educational level, marital status and average income per month.

#### Section B

Assessed the knowledge of market women on BSE practice and consisted of 13 questions with various options ranging from option A to E.

#### Section C

Assessed BC risk perception among the women. It consists of ten (10) items with a five-point Likert scale (Strongly Agree, Agree, Strongly Disagree, Disagree and Undecided).

#### Section D

Measured the practice of BSE among the market women. It contained twelve (12) multiple questions.

#### Section E

Reported information on the factors influencing the practice of BSE among the respondents. It contains eleven (11) items that are made up of ‘yes’ and ‘no’ responses.

### Validity of the instrument

The face and content validity of the instrument was established by lecturers in the Department of Nursing, Afe Babalola University, Ado-Ekiti. The questionnaire was corrected based on their suggestions.

### Reliability of the instrument

The reliability of the instrument was determined using the test–retest method. Thirty-four copies (10% of the sample size) of the instruments were administered to market women with similar characteristics at Uso. Two weeks after the initial administration of the questionnaire, another thirty-four copies were redistributed again to the same market women. The result was 0.796, which confirmed the reliability of the instrument. This indicated good internal consistency among the items on the questionnaire.

### Method of data collection

The questionnaire was administered by an interviewer. The researcher went to the study settings personally to administer the questionnaire with the help of two research assistants. The two research assistants were trained for one day by the researcher on how to administer the questionnaires. The respondents were provided detailed information on the study, and consent to participate was received.

### Method of data analysis

The collected data were assessed and tagged after collection. It was then coded and entered using Statistical Package for Social Sciences (IBM® SPSS) version 26. The data were then analysed using both descriptive and inferential statistics. Descriptive statistics such as frequencies, percentages, means and standard deviations were used. Inferential statistics were performed using chi square to test the hypotheses generated, and the level of significance was set at *P* ≤ 0.05. The results are presented in tables and figures. The results for knowledge of breast self-examination were categorized into good and poor knowledge. A score of 1 was allotted to each correct answer while ‘0’ was allotted to each wrong answer. Participants who scored ≥ 50% were categorised as those with good knowledge while those with scores < 50% were categorised into poor knowledge.

For the perceived risk, a score of 5 was allotted to responses with the highest rank (Strongly Agree) while 1 was allotted to responses with the lowest rank (Strongly Disagree). Perceived risk was categorised into low (< 50% score) and high (≥ 50% score) perceived risk The practice of breast self-examination was also categorized into poor and good practice of breast self-examination based on same classification used for knowledge of breast self-examination.

## Results

### Sociodemographic characteristics of participants

A total of 335 questionnaires were distributed and returned. The results showed that the mean age ± S.D. was 37.19 ± 9.19 years, with maximum and minimum ages of 18 and 65 years, respectively. A larger proportion of respondents were Christian (47.5%), and 60.0% of the respondents were married. A total of 47.5% were secondary school certificate holders, 15.5% had tertiary education, and more than two-thirds were from Yoruba**.** The sociodemographic characteristics of the respondents are presented in Table 2.

**Table 2:** Distribution of participants by their sociodemographic characteristics

Variables *N* = (335)	Frequency	Percentage
**Age as at last birthday: Mean: 37.19 ± 9.19**		
18–28	54	16.1
29–39	86	25.7
40–50	135	40.3
51 and above	60	17.9
**Religion**		
Christianity	159	47.5
Islam	151	45.0
Traditionalist	25	7.5
**Marital status**		
Married	204	60.9
Single	131	39.1
**Educational qualification**		
No formal education	60	17.9
Primary	64	19.1
Secondary	159	47.5
Tertiary	52	15.5
Average income per month: **Mean: 61000.00 ± 37.60**		
< ₦20,000	119	35.5
₦ 20,000 - ₦ 59,000	131	39.1
₦ 60,000 - ₦ 99,000	21	6.3
≥ ₦ 100,000	64	19.1
**Distance of your residence to the nearest health facility**		
Far	68	20.3
Moderate distance	133	39.7
Nearby	57	17.0
Immediate proximity	77	23.0
**Ethnicity**		
Yoruba	235	70.1
Hausa	30	9.0
Igbo	43	12.8
Others	27	8.1

**Fig. 1:**
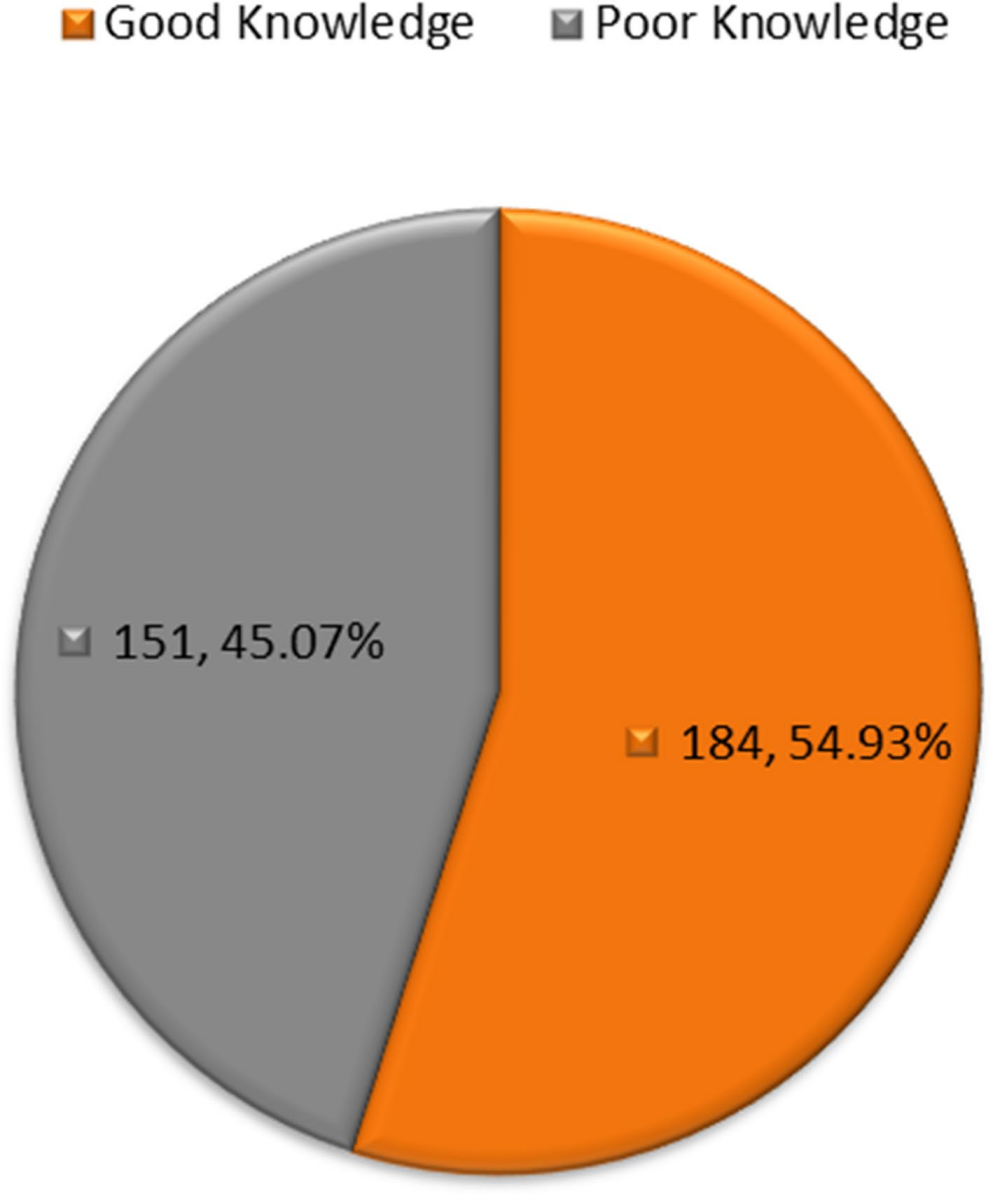
Grading of knowledge of BSE among participants

More than two-thirds (78.5%) of the participants practiced breast self-examination, 58.5% had been taught how to perform BSE, 54.3% had no knowledge that self-examination should be carried out between the 7th and 10th days of the menstrual cycle, 51.6% agreed that carrying out breast self-examination by oneself is an important strategy for detecting BC early, and 57.0% agreed that breast examinations such as mammography and CBE are carried out by clinicians. Moreover, 61.8% agreed that risk is the regular and repetitive inspection of a woman’s own breasts with the aim of detecting swelling or abnormal lumps in the breast, 69.3% agreed that breast self-examination helps in the early detection of breast changes and appearance, 78.8% agreed that breast self-examination is a quick to perform and cost-free simple procedure, 44.5% started BSE from puberty, and 46.6% of the respondents performed BSE monthly. Summarized in Table 3. The results showed that 151 (45.07%) of the respondents had poor knowledge, and a higher proportion (184, 54.93%) had a good level of knowledge of breast self-examination (Fig. 1). The perception of respondents on the risks of BC is presented in Table 4. The results showed that 75.8% of the participants agreed that the use of injectable contraception or oral pills can result in BC, and 15.8% disagreed. Similarly, 75.8% also agreed that women of reproductive age are at risk of BC. A larger proportion of respondents (75.8%) affirmed that nipple discharges, changes in breast shape and pain in the breast region are risk factors for BC, and 24.5% disagreed. A large proportion (61.2%) disagreed that the level of income in countries with low and middle income could be associated with BC, and 24.2% agreed. The results also showed that 56.4% of respondents agreed that limited knowledge of BC screening mammography clinical examination can make women underestimate the risk associated with BC, while 32.0% of respondents agreed that lobular carcinoma in situ (LCIS) is a benign condition related to BC risk but cannot lead to invasive cancer.

**Table 3:** Knowledge of breast self-examination

Variables *N* = (335)	Frequency	Percentage
**BSE can only be done by women**		
Yes	263	78.5
No	72	21.5
**I have been taught how to do BSE**		
Yes	196	58.5
No	139	41.5
**BSE should be carried out between 7 and 10**^**th**^** day of the woman menstrual cycle**		
Yes	131	39.1
No	22	6.6
I don’t know	182	54.3
**Carrying out BSE by oneself is an important strategy for detecting BC early**		
Yes	173	51.6
No	17	5.1
I don’t know	145	43.3
**Other strategies of Breast examination like mammography and CBE are carried out by clinician**		
Yes	191	57.0
No	25	7.5
No idea	119	35.5
**Risk is the regular and repetitive inspection of a woman’s own breasts with the aim of detecting swelling or abnormal lumps in breast**		
Yes	207	61.8
No	28	8.4
No idea	100	29.8
**BSE help in early detection of breast changes and appearance**		
Yes	232	69.3
No	38	11.3
I don’t know	65	19.4
**BSE is quick to perform and cost-free simple procedure**		
Yes	264	78.8
No	22	6.6
No idea	49	14.6
**Age BSE should be started**		
From birth	19	5.7
From puberty	149	44.5
From 30 years	18	5.4
From 20 years	54	16.1
After menopause	8	2.4
No idea	87	26.0
**How often BSE should be done**		
Daily	33	9.9
Weekly	61	18.2
Monthly	156	46.6
Yearly	2	0.6
No idea	83	24.7

*Key*: *BSE* Breast Self-Examination, *BC* Breast cancer, *CBE* Clinical Breast Examination

**Table 4:** Perceived risk of breast cancer

Variables *N* = (335)	Strongly Agree	Agree	Disagree	Strongly Disagree	Undecided
The use of injectable contraception or oral pills can result into BC	121(36.1)	133(39.7)	47(14.0)	6(1.8)	28(8.4)
Women of reproductive age are at risk of BC	62(18.5)	192(57.3)	54(16.1)	19(5.7)	8(2.4)
Women with large breast are at risk of BC	57(17)	106(31.6)	89(26.6)	60(17.9)	23(6.9)
Nipple discharges, change in breast shape and pain in the breast region are risk factors for BC	88(26.3)	165(49.3)	69(20.6)	13(3.9)	0(0.0)
Women who do not breastfeed their baby are at risk of BC	59(17.6)	136(40.6)	99(29.6)	24(7.2)	17(5.1)
Early onset of menarche or menopause after fifty-five years is associated with BC	11(3.3)	79(23.6)	137(40.9)	56(16.7)	52(15.5)
Giving birth to the first baby at age thirty and above could result to BC	4(1.2)	77(23)	135(40.3)	95(28.4)	24(7.2)
Level of income especially in countries with low and middle income could be associated with BC	9(2.7)	72(21.5)	120(35.8)	85(25.4)	49(14.6)
Limited knowledge of BC screening mammography clinical examination can make women to underestimate the risk associated with BC	60(17.9)	129(38.5)	52(15.5)	42(12.5)	52(15.5)
Benign that affects breast lobules is a condition related to BC risk but cannot lead to invasive cancer	23(6.9)	84(25.1)	76(22.7)	43(12.8)	109(32.5)

*Key*: *BC* Breast cancer, *BSE* Breast self-examination

### Knowledge of breast self-examination

**Fig. 2:**
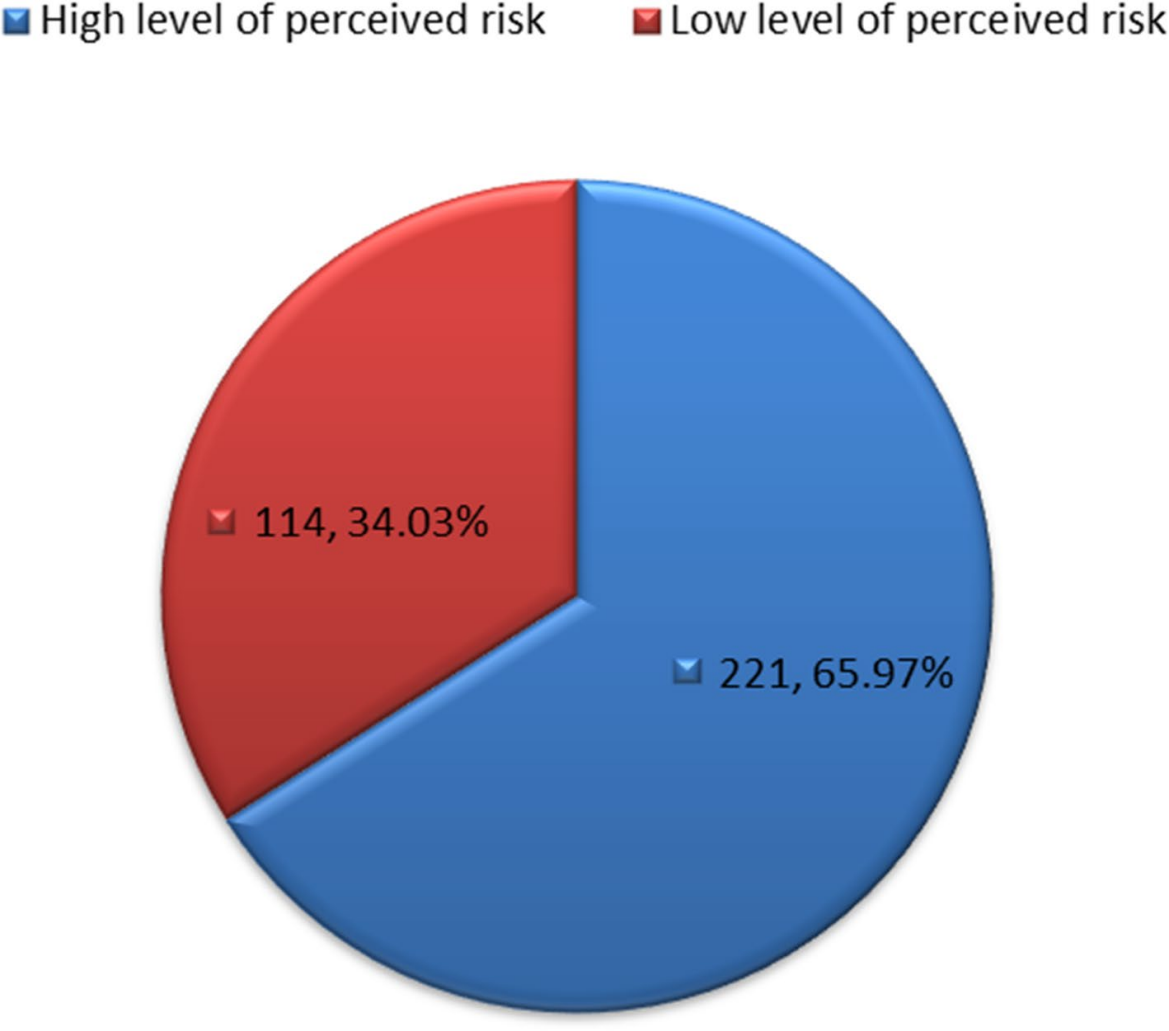
Grading of the perception of respondents on the risks of breast cancer

Assessment of the level of perceived risk of respondents about BC showed that 221 (65.97%) of respondents had a high level of perceived risk of BC, (Fig. 2).

The results showed that 69.6% of the respondents practiced BSE. It was observed that 11.9% of the respondents had performed a BSE in the last month, while 39.4% had performed a BSE in the last 2 months leading up to the study. It was observed that a major reason for some respondents not to perform BSE was lack of knowledge (16.7%) and forgetfulness (11.3%). The frequency of the performance of breast self-examination was primarily weekly (71.9%) among the respondents, and 15.5% performed it monthly. The majority, 71.6%, of the respondents performed breast self-examination while standing in front of a mirror, and 15.8% performed it by laying on a bed. Investigating how study respondents performed breast self-examination, 77.0% responded that they did so by palpating with the palm and a minimum of three fingers, and 16.7% did so by palpating with one finger. It was found that a larger proportion of the respondents, 67.5%, were not satisfied with the pattern they adopted to carry out their breast self-examination. A small fraction (5.1%) of respondents practiced BSE and found abnormalities in their breasts. The most common action that respondents mentioned as the next step whenever abnormalities appeared in their breast was taking laboratory tests (62.4%), and approximately 26.3% mentioned that they would pray. This has been summarized in Table 5.

**Table 5:** Practice of breast self-examination

Variables *N* = (335)	Frequency	Percentage
**Practice of BSE**		
Yes	233	69.6
No	102	30.4
Last time you performed BSE		
1 month ago	40	11.9
2 months ago	140	39.4
3 months ago	15	4.5
6 months ago	38	11.3
Reason for no practice of BSE		
Always forgot	38	11.3
No knowledge of it	56	16.7
Not always remember	18	5.4
**How often you perform BSE**		
Monthly	52	15.5
Weekly	241	71.9
Whenever I remember	42	12.5
Know the process and steps of BSE		
Yes	106	31.6
No	229	68.4
**When you normally perform BSE**		
Before Period	35	10.4
During period	12	3.6
After Period	64	19.1
I don’t practice	224	66.9
**Where you perform BSE**		
Lying down on the bed	53	15.8
Standing in front of the mirror	240	71.6
In the bathroom	42	12.5
**How you perform BSE**		
Palpate with one finger	56	16.7
Palpate with palm and minimum of three fingers	258	77.0
Anyhow	21	6.3
**I feel satisfied with how I perform BSE**		
Yes	109	32.5
No	226	67.5
Since I started performing BSE, I have discovered abnormalities in my breast		
Yes	17	5.1
No	105	31.3
I have not done BSE before	213	63.6
**What you will do if you discover abnormalities in your breast during BSE**		
Pray over it	88	26.3
Do some laboratory tests	13	3.9
Tests	209	62.4
See a doctor	15	4.5
Do nothing	10	3.0

*Key*: *BSE* Breast Self-Examination

**Fig. 3:**
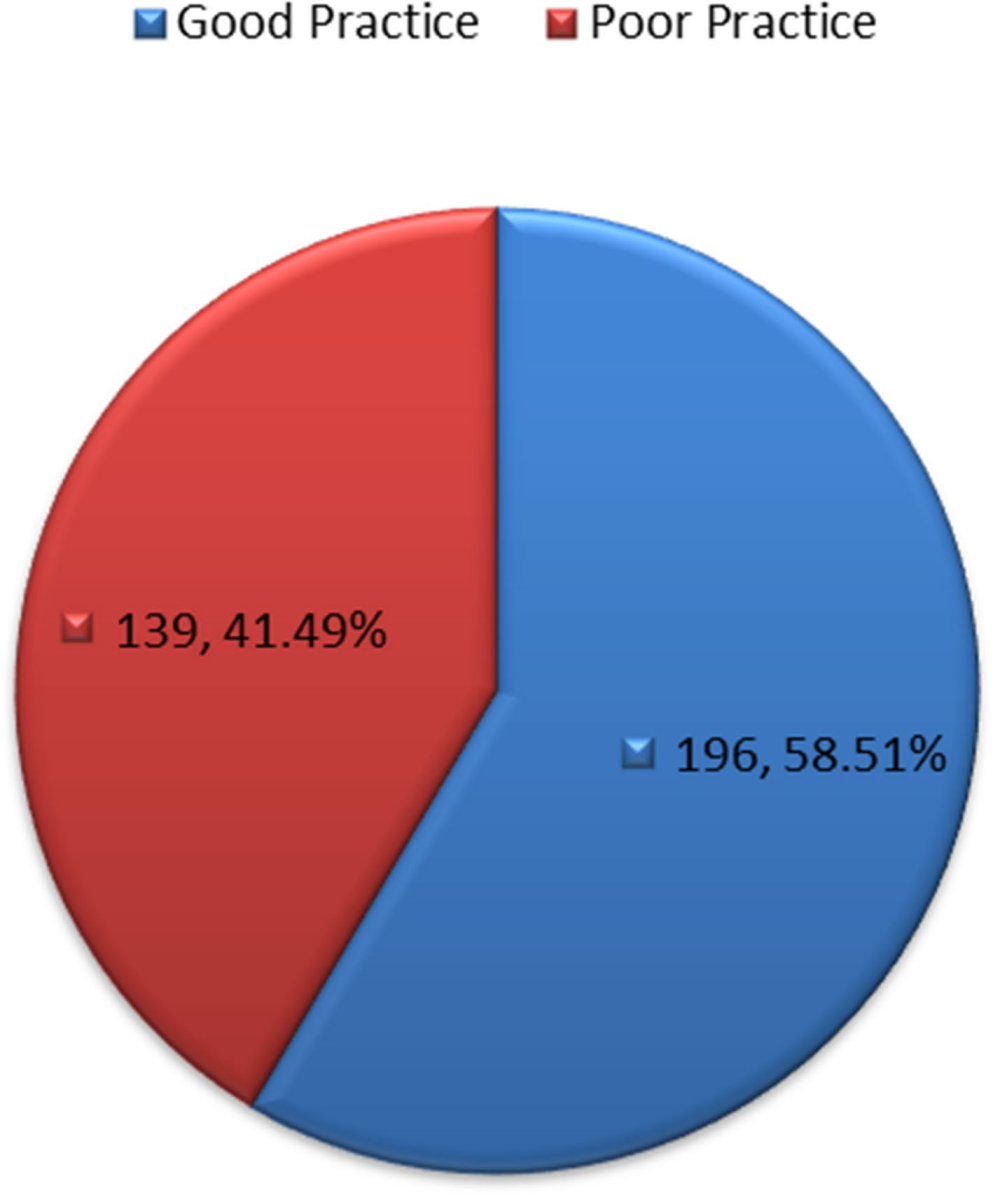
Grading of practice of BSE among participants

The level of practice of breast self-examination among respondents is presented in Fig. 3. The results showed that majority (196 (58.51%)) had a good level of practice of breast self-examination.

Factors determining the practice of breast self-examination among the respondents are presented in Table 6. It was observed that the majority, 62.7%, were not held back by fear in practising breast self-examination. Approximately 47.8% mentioned that they were uncomfortable taking off clothes in front of health professionals during the examination, and 46.3% had a feeling of being at risk of developing BC and hence did not see a need for practice of BSE. In addition, 35.5% mentioned that a lack of support from spouses, friends and family sometimes prevented them from performing BSE. Approximately 34% of respondents stated that their decisions were influenced by sociocultural milieu in the practice of BSE. The relationship between the sociodemographic characteristics of the market women and their knowledge of BSE was assessed, and it was deduced that age (*p* = 0.023), educational qualifications (*p* < 0.001), average income per month (*p* < 0.001) and ethnicity (*p* =  < 0.001) were statistically associated with knowledge of breast self-examination (Table 7). Additionally, the association between sociodemographic characteristics of respondents and perceived risk of BC showed that educational qualification (*p* = 0.001) and distance from facility (*p* = 0.009) were statistically associated with perceived risk of BC (Table 8). Participants’ educational qualification (*p* = 0.006) and ethnicity (*p* = 0.013) were statistically associated with practice of BSE (Table 9).

**Table 6:** Key influences on breast self-examination practice

Variables *N* = (335)	Yes	No
I am afraid BSE will find cancer	125(37.3)	210(62.7)
I feel uncomfortable taking off clothes in front of health professionals during the examination	160(47.8)	175(52.2)
Having BSE would be painful	71(21.2)	264(78.8)
It is against my religious beliefs to do BSE	55(16.4)	280(83.6)
My sociocultural milieu has influence in my decision to practice BSE	114(34.0)	221(66)
BSE is embarrassing	67(20.0)	268(80)
I do not know what BSE is all about	113(33.7)	222(66.3)
I do not feel at risk of getting BC so BSE is not necessary	155(46.3)	180(53.7)
Absence of support from spouse, friends and family sometimes prevent me from performing BSE	119(35.5)	216(64.5)
I have not engaged in risky sexual behaviours, so I cannot have BC	167(49.9)	168(50.1)
BC has no cure so no need of knowing my status	59(17.6)	276(82.4)

*Key*: *BC* Breast cancer, *BSE* Breast self-examination

**Table 7:** Association between sociodemographic characteristics and knowledge of breast self-examination

Variables *N* = (335)	Knowledge of BSE		X^2^	*P*
**Age as at last birthday**	**Poor**	**Good**		
18–28	25	29	9.553	0.023*
29–39	49	37		
40–50	58	77		
51 and above	19	41		
**Religion**				
Christianity	62	97	7.018	0.030*
Islam	80	71		
Traditionalist	9	16		
**Marital status**				
Married	84	119	2.608	0.106
Single	66	65		
**Educational qualification**				
No formal education	34	26	20.903	< 0.001*
Primary	38	26		
Secondary	68	91		
Tertiary	11	41		
**Average income per month**			17.009	< 0.001*
< ₦20,000	58	61		
₦ 20,000 - ₦ 59,000	61	70		
₦ 60,000 - ₦ 99,000	12	9		
≥ ₦ 100,000	7	37		
**Distance of your residence from the nearest health facility**				
Far	25	43	8.392	0.039*
Moderate distance	67	59		
Nearby	19	38		
Immediate proximity	35	42		
**Ethnicity**				
Yoruba	91	144	16.703	< 0.001*
Hausa	20	10		
Igbo	21	22		
Others	19	8		

*Key*: *Significant value when *p* ≤ 0.05, *P p*-value

**Table 8:** Association between sociodemographic characteristics and perceived risk of breast cancer

Variables *N* = (335)	Perceived risk of Breast cancer		X^2^	*P*
**Age as at last birthday**	**Low**	**High**		
18–28	18	36	1.807	0.613
29–39	28	58		
40–50	51	84		
51 and above	17	43		
**Religion**				
Christianity	62	97	3.400	0.183
Islam	44	107		
Traditionalist	8	17		
**Marital status**				
Married	72	131	0.411	0.521
Single	42	89		
**Educational qualification**				
No formal education	13	47	22.564	< 0.001*
Primary	20	44		
Secondary	49	110		
Tertiary	32	20		
**Average income per month**			2.337	0.505
< ₦20,000	37	82		
₦ 20,000 - ₦ 59,000	52	79		
₦ 60,000 - ₦ 99,000	8	13		
> ₦ 100,000	14	30		
**Distance of your residence from the nearest health facility**				
Far	21	47	11.598	0.009*
Moderate distance	57	69		
Nearby	17	40		
Immediate proximity	18	59		
**Ethnicity**				
Yoruba	86	149	2.480	0.479
Hausa	8	22		
Igbo	13	30		
Others	7	20		

*Key*: *Significant value when *p* ≤ 0.05, *P p*-value

**Table 9:** Association between sociodemographic characteristics and practice of breast self-examination

**Variables *N* = (335)**	**Practice of BSE**		**X**^**2**^	***P***
**Age as at last birthday**	**Poor**	**Good**		
18–28	22	32	2.283	0.516
29–39	34	52		
40–50	62	73		
51 and above	21	39		
**Religion**				
Christianity	62	97	2.580	0.275
Islam	63	88		
Traditionalist	14	11		
**Marital status**				
Married	76	127	3.212	0.073
Single	62	69		
**Educational qualification**				
No formal education	31	29	19.860	< 0.001*
Primary	33	31		
Secondary	67	92		
Tertiary	8	44		
**Average income per month**			12.586	0.006*
< ₦20,000	55	64		
₦ 20,000 - ₦ 59,000	53	78		
₦ 60,000 - ₦ 99,000	8	13		
≥₦ 100,000	7	37		
**Distance of your residence from the nearest health facility**				
Far	24	44	3.551	0.314
Moderate distance	56	70		
Nearby	19	38		
Immediate proximity	35	42		
**Ethnicity**				
Yoruba	95	140	10.759	0.013*
Hausa	14	16		
Igbo	12	31		
Others	18	9		

*Key*: *Significant value when *p* ≤ 0.05, *P p*-value

There was a higher likelihood of having good knowledge of BSE among participants aged 40–50 years (0.764 (0.320–1.826); *p* = 0.545) and those aged ≥ 51 years (2.062 (0.729–5.830); *p* = 0.172) compared to those aged 18–28 years. There was also a higher likelihood in showing good practice of BSE among women aged 29–39 years (0.652 (0.258–1.644); *p* = 0.364) and ≥ 51 years (1.317 (0.505–3.436); *p* = 0.573) than those aged 18–28 years (Table 10).

**Table 10:** Regression analysis between demographics, knowledge, perceived risks and practice of breast self-examination among study participants

Variables		Good knowledge of BSE		High perceived risk of BC		Good practice of BSE	
		**Odds Ratio (95% CI)**	***P*-value**^**a**^	**Odds Ratio (95% CI)**	***P*-value**^**a**^	**Odds Ratio (95% CI)**	***P*-value**^**a**^
Age (yrs) (**Ref: 18–28)**	29–39	0.251 (0.091–0.692)	0.008*	**0.390 (0.159–0.954)**	0.039*	0.652 (0.258–1.644)	0.364
	40–50	0.764 (0.320–1.826)	0.545	0.471 (0.173–1.285)	0.142	0.453 (0.194–1.056)	0.067
	51 and above	2.062 (0.729–5.830)	0.172	0.592 (0.223–1.571)	0.293	1.317 (0.505–3.436)	0.573
Religion (**Ref: Christianity**)	Islam	0.692 (0.358–1.262)	0.217	1.893 (1.026–3.495)	0.041*	1.515 (0.831–2.759)	0.175
	Traditionalist	9.105 (2.503–33.120)	< 0.001*	1.500 (0.494–4.556)	0.475	1.609 (0.544–4.760)	0.390
Married (**Ref: single**)		0.348 (0.177–0.683)	0.002*	0.881 (0.477–1.627)	0.686	0.506 (0.276–0.929)	0.028*
Education (**Ref: No formal Education**)	Primary	0.719 (0.275–1.884)	0.502	0.575 (0.230–1.439)	0.237	0.972 (0.421–2.247)	0.947
	Secondary	2.969 (1.323–6.662)	0.008*	0.663 (0.298–1.471)	0.312	2.097 (1.018–4.316)	0.044*
	Tertiary	6.601 (2.350–18.544)	< 0.001*	0.180 (0.069–0.475)	< 0.001*	5.209 (1.906–14.233)	0.001*
Income (**Ref: < N 20,000**)	N 20,000—N 59,000	0.656 (0.357–1.207)	0.176	0.606 (0.336–1.096)	0.098	0.895 (0.509–1.573)	0.700
	N 60,000—N 99,000	0.486 (0.147–1.606)	0.237	0.631 (0.194–2.044)	0.442	1.388 (0.417–4.618)	0.593
	> N 100,000	4.729 (1.548–14.448)	0.006*	1.356 (0.538–3.420)	0.519	3.493 (1.294–9.428)	0.014*
Distance (**Ref: Far**)	Moderate	1.913 (0.878–4.167)	0.102	2.613 (1.245–5.483)	0.011*	1.226 (0.589–2.552)	0.586
	Nearby	3.459 (1.335–8.963)	0.011*	2.407 (1.046–5.540)	0.039*	1.462 (0.626–3.413)	0.380
	Immediate proximity	2.033 (1.001–4.132)	0.050*	2.552 (1.260–5.172)	0.009*	0.902 (0.464–1.752)	0.760
Good knowledge of BSE (**Ref: Poor**)		-	**-**	-	**-**	4.574 (2.841–7.365)	< 0.001*
High perceived risk of BC (**Ref: Low**)		-	**-**	-	**-**	0.765 (0.458–1.276)	0.305

*Key*: *CI* Confidence interval, *BC* Breast Cancer, *BSE* Breast Self-examination

^a^Binary logistic Regression

^*^Statistically significant value at *p* ≤ 0.05

Participants who were married showed significantly lower likelihood to have good knowledge of BSE than single (0.348 (0.177–0.683); *p* = 0.002). However, there was a significantly higher likelihood to having good practice of BSE (0.506 (0.276–0.929); *p* = 0.038) and non-significantly higher likelihood to have a high perceived risk of BC (0.881 (0.477–1.627); *p* = 0.686) among married participants than single (Table 10).

There was increasing likelihood in showing good knowledge (Secondary: 2.969 (1.323–6.662), *p* = 0.008; Tertiary: 6.601 (2.350–18.544), *p* < 0.001) and good practice of BSE (Secondary: 2.097 (1.018–4.316), *p* = 0.044; Tertiary: 5.209 (1.906–14.233), *p* = 0.001) among participants with improved educational status than those with no formal education.

The women earning ₦ 20,000 - ₦ 59,000 showed a higher likelihood to having good knowledge of BSE (0.656 (0.357–1.207), *p* = 0.176), high perceived risk of BC (0.606 (0.336–1.096), *p* = 0.098) and good practice of BSE (0.895 (0.509–1.573), *p* = 0.700) than those earning < ₦ 20,000. There was significantly higher likelihood of good knowledge (4.729 (1.548–14.448), *p *= 0.006) and good practice of BSE (3.493 (1.294–9.428), *p* = 0.014) among those earning > ₦ 100,000 than those earning < ₦ 20,000.

Participants who stayed nearby (3.459 (1.335–8.963), *p* = 0.011) and within immediate proximity (2.033 (1.001–4.132), *p* = 0.050) had a significantly higher likelihood to showing good knowledge of BSE than those living far from the facility. There was also significantly higher likelihood of showing high perceived risk of BC in participants living at moderate (2.613 (1.245–5.483), *p* = 0.011), nearby (2.407 (1.046–5.540), *p* = 0.039) and immediate proximity (2.552 (1.260–5.172), *p* = 0.009) than those living far from the facility.

Participants with good knowledge showed a significantly higher likelihood of having good practice of BSE (4.574 (2.841–7.365), *p* < 0.001). Also, high perceived risk of BC was associated with a higher likelihood of good practice of BSE among the participants (0.765 (0.458–1.276), *p* = 0.305). Other information has been summarised in Table 10.

## Discussion

### Sociodemographic characteristics of respondents

Globally, BC is the most common cancer. It has been proven that early detection and treatment of the disease lowers mortality rates [17, 18]. Data were collected from women in the selected markets, and the age distribution of women in this study was found to have a mean ± SD of 37.19 ± 9.19 years and a range from 18 to 65 years old. This result is corroborated by the findings of Kumarasamy et al. (2017) in Tamil Nadu [19] and higher than those reported by Dadzi & Adam (2019) among women in the Southern District of the Volta region of Ghana [20]. In addition, Jothula & Sreeharshika. (2020), reported a higher mean age of 40.7 ± 9.5 years for rural women in Telangana [21]. According to Kedir et al. (2021), BSE is a necessary option for women in their 20 s and older [22]. The study findings showed a higher proportion of Yoruba women participating in the study, and the majority had formal education, were married and earned approximately 50,000 naira or less monthly. Dadzi and Adam (2019) reported that the majority of the women present in their study were single, and only 3.1% had no formal education [20].

### Knowledge of breast self-examination

BSE has been identified as one of the early detection tools for BC. According to the findings of this study, the majority of the participants had been taught how to perform BSE and performed BSE. This was found to be higher than reports among university students in Ethiopia [22], Nigeria [23], and India [24]. Moreover, this finding was observed to be higher than that of some other studies conducted among the female population. This study finding showed that there has been improvement in the knowledge of women about BSE against the report of Gwarzo et al. (2009), where less than 20% of women were reported to know about BSE [24]. Moreover, Jothula and Sreeharshika (2020) reported a low level of awareness and knowledge among rural women in Telangana [21].

The results further showed that 69.3% of women knew that BSE helps in the early detection of BC, 78.8% had knowledge of the cost implications of performing BSE, and 48.8% had knowledge that it must be performed monthly. This result is higher than the report of Nde et al. (2015) conducted among undergraduate students in Buea, where it was found that 37.3% knew that BSE was to be conducted monthly [25]. Furthermore, this finding was higher than that in a report by Kumarasamy et al. (2017) about rural women, where it was discovered that only 14% knew that BSE was to be conducted monthly [19]. Another study conducted among rural women reported a prevalent low level of knowledge of BSE in Telangana [21].

Knowledge of the women about BC and BSE serves as a measure to detect BC early and to practice BSE correctly. This study showed that 45.07% had poor knowledge about BSE. This is higher to the report of Bellgam & Buowari (2012), where 36.5% showed poor knowledge [26]. However, the findings of this result are lower when compared with the students in college girls in Udupi district (72.5%) with good knowledge, as reported by Shalini et al. (2011) [27]. The possible reason for this could be that this study was carried out among market women with majority having at least secondary education. The level of knowledge of BSE found in the present study is similar to a report by Oladimeji et al. (2015) among women in Ibadan, Southwest Nigeria [28].

The association between sociodemographic variables and knowledge of the BSE showed that age, educational qualifications, average monthly income, and distance from facility were factors that were significantly associated with market women’s knowledge of the BSE. Also, study participants with higher educational qualifications showed a higher likelihood of having good knowledge of BSE. This result corroborates the findings of Dewi et al. (2019), who reported that well-educated women were more knowledgeable about breast self-examination [29]. This highlights the importance of education to improved knowledge of BSE.

Similarly, good knowledge of BSE was associated with increased age among the participants. This is in line with findings in Southwest Nigeria where there was a significant difference in knowledge among age groups of the market women with women in higher age groups showing higher knowledge scores [28]. This shows that increased age is an important predictor of good knowledge of BSE.

### Perceived risk of breast cancer

The present study evaluated the risk perception of women about BC. It was believed that a woman's perception of her risk of getting BC may lead her to take steps that may protect her from getting the disease. The perceived threat of disease, when a woman perceived the risk of getting BC, will lead her to take steps that may protect her from getting the disease. The results showed that 75.8% of the participants agreed that the use of injectable contraception or oral pills can result in BC. This corroborates the findings in Denmark, where it was reported that the risk of BC increased with women using hormonal contraceptives and further increased with increased duration of use [30].

Additionally, the present study found that 75.8% of women further asserted that women of reproductive age are at risk of BC and that nipple discharges, changes in breast shape and pain in the breast region are risk factors for BC. Breastfeeding and BC have a contentious relationship. Breastfeeding over 12 months reduced the incidence of BC by 4.3 percent, according to a review by Ozsoy et al. (2017), which included 30 nations, 47 epidemiological studies, 50302 BC and 96973 non-BC patients [31]. This study found that more than an average proportion of the respondents had low perception or knew that breastfeeding was a risk factor for BC.

In the present study, 26.9% of women agreed that early onset of menarche or menopause after fifty-five years is associated with BC. This was not in tandem with a study by Ozsoy et al. (2017) among women who had mammography (MG) for BC screening [31]. It has been estimated that every year of delay after the age of 12 reduces the premenopausal BC risk by 7% and postmenopausal cancer by 3% [32].

### Breast self-examination practice

This study found that the majority, 58.51%, of women in the study area had good practice of BSE. The results showed that nearly seven out of ten of the women present in this study engaged in BSE. It was also found that approximately 11.9% performed it within the last month prior to this study, and 39.4% practiced it within 2 months prior to this study. This degree of practice is higher than that reported in Malaysia [24] and Cameroon [25, 33]. Dewi et al. (2019) found that among 2173 women in Indonesia, only 44.4% indicated that they had previously performed BSE [29]. This study finding was lower than that reported by Florence et al. (2016), who estimated that 85%—90% of students perform self-breast examinations monthly [34]. This buttresses the importance of knowledge and education on the practice of BSE. Lack of knowledge, according to Nde et al. (2015) [25], Suh et al. (2012) [33], and Gwarzo et al. (2009) [24], is associated with a low level of BSE practice. This statistic is also greater than a previous survey conducted in Benin, Nigeria, which found that 50% of women practiced BSE [35]. Doshi et al. (2012) revealed in their study that the practice of BSE among a group of Indian dental students was alarmingly low [36]. It is possible that the low prevalence of BSE among earlier participants was due to research focusing on rural/market or less educated women who were unaware of the benefits of BSE. The present study established that the major hindrances to the practice of BSE were religious beliefs, feelings such as discomfort in removing clothes, and embarrassing acts of touching the breast. Other factors include sociocultural milieu and incorrect knowledge about BSE, such as breast self-examination, which could be a painful, incurable disease. According to Kumar and Kashyap (2016), lack of knowledge, embarrassment, and fear are reasons for not performing BSE, which corroborates the present study findings [37].

The present study found that higher perceived risk of BC and good knowledge of BSE is associated with good practice of BSE. Dadzi, & Adam. (2019) likewise found that knowledge of BSE is a significant predictor of BSE practice [20]. This showed significantly that women’s decision to practice BSE is strongly influenced by their level of risk perception about BC. It is worth noting that Nde et al. (2015) [25] and Agbonifoh (2016) [35] reported that the incidence of a family history of BC was strongly related to the actual practice of BSE among study participants. A negative relationship between age and the practice of BSE was established by Dadzi & Adam. (2019) among women in the Volta region of Ghana, where it was established using a linear regression model that the level of BSE practice among participants decreased as they became older [20]. Moreover, the odds of practicing BSE increased, and the results demonstrated that a woman's knowledge of BC and BSE are major predictors of BSE. There were significant associations between knowledge, level of education and practice of BSE in a study conducted among women of reproductive age in Ibadan, Nigeria [38].

Furthermore, it was shown that the income status of participants and closer proximity to facility directly affected their practice of BSE. Azemfac and colleagues reported that women who lack of proximity to formal medical care were less likely to have good practice of BSE [39]. Also, income limits play an essential role in access to the various resources of information regarding BC screening practice according to a similar study [40].

### Limitations of the study

This study was limited to selected markets in Owo town; hence, it cannot be generalized; nevertheless, this study was conducted vigorously and will contribute immeasurably to the health of women in the Owo local government area. The design effect was not applied in the calculation of sample size because intra-cluster correlation required for actual estimation of the sample size could not be determined as at the time of the study. While this could affect the actual sample size calculated, the findings of this study is still valuable. Further studies are recommended to assess the factors responsible for the inconsistency of BSE using participatory observational studies and in-depth interviews and focus group discussions that will cut across the three senatorial districts of Ondo state.

## Conclusion

This study identified high level of perception, good knowledge and good practice of BSE among majority of the market women in Owo Town. The practice has been linked with some important predictive factors. It is therefore important to implement interventions and extensive health education or campaigns on BSE with the aim of creating positive awareness and understanding of BSE among the population. This would minimize the rate of late detection of new cases as well as reduce continuous morbidity and mortality linked with BC.

## Data Availability

Data material used in this study was confidential and will be made available by the corresponding authors on request.

## Abbreviations

BC: Breast cancer
BSE: Breast self-examination
CBE: Clinical breast examination
LCIS: Lobular carcinoma in situ
LMICs: Low- to medium-income countries
WHO: World Health Organization
